# Response: Commentary: Revised contraindications for the use of non-medical WB-electromyostimulation. Evidence-based German consensus recommendations”

**DOI:** 10.3389/fspor.2024.1473103

**Published:** 2024-10-21

**Authors:** W. Kemmler, M. Fröhlich, O. Ludwig, C. Eifler, J. Berger, F. Micke, H. Kleinöder, B. Wegener, C. Zinner, F. C. Mooren, M. Teschler, A. Filipovic, S. Müller, K. England, J. Vatter, S. Authenrieth, M. Kohl, S. von Stengel

**Affiliations:** ^1^ Institute of Radiology, University Hospital Erlangen, Erlangen, Germany; ^2^ Department of Sports Science, Rheinland-Pfälzische Technische Universität Kaiserslautern-Landau, Kaiserslautern, Germany; ^3^ German University for Prevention and Health Management, Saarbrücken, Germany; ^4^ Institute of Training Science and Sport Informatics, German Sport University Cologne, Cologne, Germany; ^5^ Musculoskeletal University Center, Ludwig-Maximilian-University of Munich, Munich, Germany; ^6^ Hessian College of Police and Administration, Wiesbaden, Germany; ^7^ Department of Rehabilitation Sciences, Faculty of Health, University of Witten/Herdecke, Witten, Germany; ^8^ Soccer Club Paderborn 07, Paderborn, Germany; ^9^ Glucker Kolleg, Frankfurt, Germany; ^10^ Bundeswehr Medical Academy Munich, Munich, Germany; ^11^ PT Lounge Cologne, Cologne, Germany; ^12^ EMS-Performance, Kornwestheim, Germany; ^13^ Department of Medical and Life Sciences, University of Furtwangen, Schwenningen, Germany

**Keywords:** whole-body electromyostimulation, contraindications, safety, exercise, oncology, tumor, cancer

A Commentary on Commentary: Revised contraindications for the use of non-medical WB-electromyostimulation. Evidence-based German consensus recommendations By Reljic D, Herrmann HJ and Zopf Y (2024). Front Sports Act Living. 6:1425233. doi: 10.3389/fspor.2024.1425233

## Introduction

There is an ongoing discussion on safety aspects related to the commercial whole-body electromyostimulation (WB-EMS) market. Apart from mandatory federal regulation, the most controversial area may well be the relative and absolute contraindications for WB-EMS application. In a recent commentary, Reljic et al. (1) addressed the “revised contraindications for the use of non-medical WB-Electromyostimulation adopted as an evidence based consensus recommendation by a broad consortium of German researchers (2). In summary, Reljic et al. (1) criticize in particular the current status of tumor and cancer still being classified as a relative contraindication for commercial, non-medical WB-EMS application. Considering the outstanding experience of this research group in the evaluation of WB-EMS application in cancer patients (3), we take their objection very seriously and would like to explain our decision in more detail.

First of all, we feel that due to our strict evidence- and consensus-based approach, some basic aspects might have not pointed out clearly enough. In line with Reljic et al, we fully agree that WB-EMS is a very safe exercise technology when properly applied (3, 4). So far, apart from a few trials that provoke rhabdomyolysis by excessive impulse intensity in novice applicants [e.g., (5, 6)], no clinical trial has reported acute or chronic “suspected unexpected serious adverse reaction” (SUSARs) during or after WB-EMS application (3, 4, 7). However, the safety standards of clinical studies with their trial physicians, study nurses, experienced trainers and scientific monitoring boards vary considerably from the real world setting of commercial, non-medical suppliers. As a matter of fact, the present strategy of commercial facilities of training up to 200 widely different clients per week in parallel on two WB-EMS devices supervised by one instructor cannot but conflict with the need to consistently handle vulnerable cohorts with adequate care und expertise. Thus, we are convinced that our contraindications that focus exclusively on commercial non-medical WB-EMS settings protect not only vulnerable clients but also over motivated trainers and owners of WB-EMS facilities from themselves. To stress the above aspect again, people with conditions contraindicated to WB-EMS application in commercial, non-medical facilities were not excluded from each and every WB-EMS application. On the contrary, medical WB-EMS facilities (8)^1^ with their enhanced medical expertise and closer supervision are perfectly suited for applying this safe and efficient exercise technology in vulnerable cohorts. This approach of well-controlled medical settings is fully in line with the demand of Reljic et al. for appropriate medical settings for tumor patients.

Another source of misunderstanding might be our interpretation of absolute and relative contraindications. In general, contraindications predominately emphasize the balance between risks and benefits of a treatment or procedure. An absolute contraindication was considered as an event or substance that could cause a life-threatening situation. Correspondingly, a procedure or medicine that falls under this category must be avoided (9). However, particularly the risk of physical exercise including WB-EMS in vulnerable people depends greatly on the framework of its application. In a closely supervised and experienced medical WB-EMS setting, the risk of adverse effects or even life-threatening situations is much less pronounced for vulnerable cohorts compared to commercial, non-medical settings. This aspect leads to diverging results of risk/benefit assessments in medical vs. non-medical WB-EMS facilities, and ultimately to the outcome that the absolute contraindications for commercial non-medical facilities listed in the “revised contraindications” (2) are not necessarily applicable for medical WB-EMS institutions. Considering further that even relative contraindications still need a medical risk/benefit assessment prior to approval, we feel that the switch of tumor and cancer from an absolute to a relative contraindication for commercial non-medical WB-EMS application is a reasonable and responsible step to easily enable the application of WB-EMS in stable tumor patients, for example. In contrast to the demand of Reljic et al. (1), however, this means that after medical approval, eligible tumor patients are enabled to exercise in non-medical facilities.

With reference to the recommendation of Reljic et al. (1) to base relative contraindication for tumor and cancer on the more specific oncologic exercise guideline, we have also considered this dedicated and precise approach in the consensus conferences. Nevertheless, since exercise guidelines are also available for other diseases (e.g., diabetes mellitus) covered in this publication on WB-EMS contraindications (10, 11), listing all condition-specific contraindications would not be compatible with a guideline that is still easy to apply. However, the aspect that potential relative contraindications have to be verified by the attending physician aware of the limitation of their patient will ensure the application of dedicated-condition specific recommendations.

Another critical aspect related to the proper application of contraindications is the role of the medical gate keeper (especially physicians) and the availability of medical WB-EMS. Despite the large popularity and widespread distribution of WB-EMS in Germany, the necessary WB-EMS expertise of the medical decision-maker cannot be universally assumed, which might lead to unnecessary decisions against WB-EMS application. Further, the presently decelerated dissemination of medical WB-EMS prevents reaching vulnerable cohorts that could particularly benefit from WB-EMS. This development is due not least to the limited prescribability of WB-EMS by the healthcare system.

Finally one should keep in mind the non-mandatory character of our “revised contraindications for WB-EMS application”. But having said that, in the light of bans (12), critical public media releases (13) and serious federal regulations (14, 15), the safety aspects on WB-EMS should be given maximum priority in order to prevent further restrictions. One may consider the particular negative focus on WB-EMS compared to conventional types of exercise as unfair. Nevertheless, it should be noted that the simultaneous stimulation of (all) large muscle areas, the possibility of applying supramaximal stimulus intensity and especially the aspect that protective physiological mechanisms of muscle fatigue and overload (16) do not apply to NEMS techniques and so constitute an enhanced risk potential. Correspondingly a close attention with safety aspects to WB-EMS application is well advised.

## Footnotes

^1^Medical WB-EMS is defined as a (1) primarily therapeutic intervention (2) based on an existing diagnosis (3) that is provided by qualified medical–therapeutic personnel (4) in compliance with current guidelines and (5) using medical devices (8).
